# Epac1 links prostaglandin E_2_ to β-catenin-dependent transcription during epithelial-to-mesenchymal transition

**DOI:** 10.18632/oncotarget.10128

**Published:** 2016-06-17

**Authors:** Sepp R. Jansen, Wilfred J. Poppinga, Wim de Jager, Frank Lezoualc'h, Xiaodong Cheng, Thomas Wieland, Stephen J. Yarwood, Reinoud Gosens, Martina Schmidt

**Affiliations:** ^1^ Department of Molecular Pharmacology, Groningen Research Institute for Pharmacy (GRIP), University of Groningen, Groningen, The Netherlands; ^2^ Inserm UMR-1048, Institut des Maladies Métaboliques et Cardiovasculaires, Université Toulouse III, Toulouse, France; ^3^ Department of Integrative Biology & Pharmacology, Texas Therapeutics Institute, Brown Foundation Institute of Molecular Medicine, University of Texas, Houston, TX, USA; ^4^ Institute of Experimental and Clinical Pharmacology and Toxicology, Medical Faculty Mannheim, University of Heidelberg, Heidelberg, Germany; ^5^ School of Life Sciences, Heriot-Watt University, Edinburgh, Scotland

**Keywords:** PGE_2_, Epac, β-catenin, Ezrin, EMT

## Abstract

In epithelial cells, β-catenin is localized at cell-cell junctions where it stabilizes adherens junctions. When these junctions are disrupted, β-catenin can translocate to the nucleus where it functions as a transcriptional cofactor. Recent research has indicated that PGE_2_ enhances the nuclear function of β-catenin through cyclic AMP. Here, we aim to study the role of the cyclic AMP effector Epac in β-catenin activation by PGE_2_ in non-small cell lung carcinoma cells.

We show that PGE_2_ induces a down-regulation of E-cadherin, promotes cell migration and enhances β-catenin translocation to the nucleus. This results in β-catenin-dependent gene transcription. We also observed increased expression of Epac1. Inhibition of Epac1 activity using the CE3F4 compound or Epac1 siRNA abolished the effects of PGE_2_ on β-catenin. Further, we observed that Epac1 and β-catenin associate together. Expression of an Epac1 mutant with a deletion in the nuclear pore localization sequence prevents this association. Furthermore, the scaffold protein Ezrin was shown to be required to link Epac1 to β-catenin.

This study indicates a novel role for Epac1 in PGE_2_-induced EMT and subsequent activation of β-catenin.

## INTRODUCTION

Non-small cell lung carcinoma (NSCLC) is the most common variety of lung cancer and is the leading cause of cancer related deaths worldwide. One of the major causes for mortality is dissemination [1, 2] whereby, early in the metastatic cascade, carcinoma cells gradually lose their epithelial phenotype and acquire a motile, mesenchymal phenotype. This process is known as epithelial-to-mesenchymal transition (EMT) [3]. In NSCLC cells, EMT can be induced by a variety of growth factors and other molecular mediators, including prostaglandin E_2_ (PGE_2_) [4–8]. PGE_2_ is a potent inflammatory mediator produced by cyclooxygenase-2 (COX-2), whose levels are often found to be increased in many cancers, including lung carcinoma [9]. Elevated levels of PGE_2_ have been found to contribute to the induction of tumor angiogenesis, resistance to apoptosis, regulation of cell division, suppression of anti-tumor immunity and augmentation of cancer cell motility and invasiveness [10]. The use of selective COX-2 inhibitors has raised multiple concerns regarding its safety of long-term use in clinical trials [11]. Thus, further exploration of the pathways downstream of PGE_2_ receptor binding is important to elucidate the mechanisms of PGE_2_ signaling in cancer and could provide the basis for the development of novel therapy.

Previous work from our group and others has shown that PGE_2_ results in the stabilization of the important oncogene β-catenin in cancer cells [12–14]. β-Catenin has a dual function in the cell; on one hand it is a component of the adherens junction, where it stabilizes E-cadherin/actin cytoskeleton binding, on the other hand, free cytosolic β-catenin can translocate to the nucleus. Although free cytosolic β-catenin levels are normally under tight control, accumulation of stabilized β-catenin eventually leads to its nuclear translocation, where it acts as a transcriptional co-regulator. As such, nuclear β-catenin activates transcription of a broad range of target genes involved in survival and metastasis [15, 16]. In an *in vivo* model of colorectal carcinoma, it has been demonstrated that nuclear β-catenin and subsequent activation of TCF, a transcription factor commonly associated with nuclear β-catenin, increases the expression of the important EMT transcription factor zinc finger E-box binding homeobox 1 protein (ZEB1) [17], of which the expression has the most consistent inverse correlation with E-cadherin expression across different types of carcinomas [18]. This mechanism was recently confirmed in a pancreatic cancer model [19] and in an *in vivo* kidney model for EMT [20]. Thus, activation of β-catenin/TCF-dependent transcription (referred to as β-catenin-dependent transcription) can induce EMT, thereby down-regulating E-cadherin expression, further releasing β-catenin form the adherens junction, creating a positive feedback loop that attenuates cell-cell adhesion and reinforces EMT in transformed cells. The existence of this loop has been confirmed in a breast cancer stem cell model in which inhibition of β-catenin, using the β-catenin/p300 inhibitor curcumin, breaks the loop, restoring E-cadherin expression and sequestering β-catenin at cell-cell contacts [21]. In NSCLC cells, PGE_2_ has been found to induce EMT and enhance cell migration by augmenting ZEB1 and suppressing E-cadherin expression [4–8] via a mechanism requiring stabilization of β-catenin and activation of β-catenin-dependent transcription [4, 7, 8].

PGE_2_ exerts it's intracellular actions by binding to membrane bound E-type prostanoid receptors, of which type 2 and type 4 are known to couple to Gα_s_ and thereby increase intracellular cyclic AMP. There are two known effectors of cyclic AMP; namely protein kinase A (PKA) and exchange protein directly activated by cyclic AMP (Epac). There are two Epac isoforms, Epac1 and Epac2, which have distinct tissue expression patterns [22]. In addition, Epac activity is regulated through interaction with other intracellular proteins, such as Ezrin-radixin-moesin (ERM) proteins at the cell membrane [23–25] and the nucleoporin, Ran binding protein 2 (RanBP2), at the nuclear membrane [26–29]. Interestingly, a body of recent evidence indicates that Epac is required for cancer cell migration [30–36].

Here, we aim to study the contribution of Epac to PGE_2_ and β-catenin-induced EMT and cell migration in NSCLC cells.

## RESULTS

### PGE_2_ induces epithelial-to-mesenchymal transition

**Figure 1:**
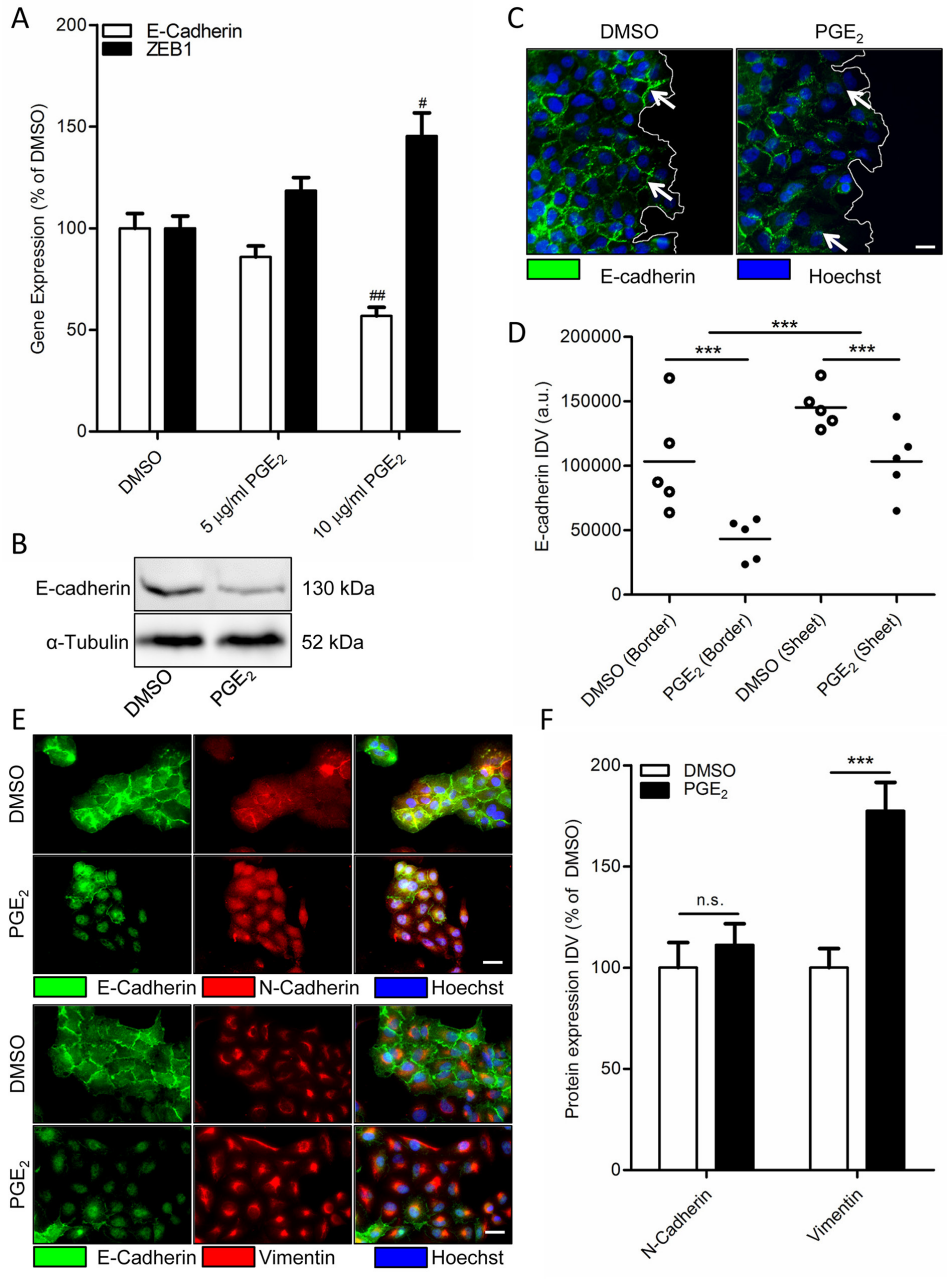
Effect of PGE_2_ on EMT in A549 cells **A.** Gene expression of E-cadherin and ZEB1 following 18 hours stimulation with PGE_2_ (10 μg/ml). **B.** Representative western blot image of E-cadherin expression in a subconfluent culture of A549 cells stimulated for 18 hours with PGE_2_. **C.** Immunofluorescence images of E-cadherin after18 hours stimulation with PGE_2_. The white line indicates the migrating border in a scratch wound assay. White arrows in indicate areas of cell-cell contact, which are decreased in cells on the migrating border in the right image. Scale bar represents 20 μm. **D.** Quantification of E-cadherin expression in migrating border cells and cells incorporated in an epithelial sheet. Each points represents the average integrated density value (IDV) of 20 cells. **E.** Immunofluorescence images of N-cadherin/E-cadherin and Vimentin/E-cadherin after18 hour stimulation with PGE_2_. Scale bar represents 20 μm. Data represent mean ± SEM of 5 separate experiments. # p < 0.05, ## p < 0.01 compared to DMSO treated cells. *** p < 0.001 between the indicated groups. **F.** Quantification of N-cadherin and Vimentin expression in cells treated for 18h with DMSO or PGE_2_.

In multiple cancer cell models, including NSCLC cells, PGE_2_ has been found to induce EMT [4, 5, 7, 8, 41]. To study the role of PGE_2_ in NSCLC, we used A549 as a cell model, which is of alveolar epithelial origin. To confirm PGE_2_-induced EMT in A549 cells, cells were incubated with 16,16-dimethyl-PGE_2_ (PGE_2_) for 18 hours. Subconfluent cultures showed decreased mRNA and protein expression of the epithelial marker E-cadherin after PGE_2_ treatment (Figure 1A-1B). Expression of the important regulatory EMT transcription factor and β-catenin target gene, ZEB1, was found to be increased by PGE_2_ treatment (Figure 1A). Interestingly, after scratch-wounding of a confluent monolayer, PGE_2_ treatment resulted in decreased E-cadherin protein expression, primarily in cells on an edge, while cells that were fully incorporated in the epithelial structure were less affected (Figure 1C-1D). In addition, immunofluorescence staining revealed that PGE_2_ does not increase overall expression of the mesenchymal marker N-cadherin, while intracellular distribution is altered with N-cadherin being less present at the cell membrane (Figure 1E-1F). However, expression of the mesenchymal marker vimentin was increased. This confirms PGE_2_ as an EMT inducer in A549 cells that are not fully incorporated in an epithelial structure.

### PGE_2_ enhances β-catenin nuclear translocation and β-catenin-dependent transcription

E-cadherin and β-catenin are both present in multiprotein complexes, known as the adherens junctions, which mediate cell-cell contacts. When E-cadherin is downregulated, β-catenin can diffuse freely in the cytosol and nucleus and promote gene expression [16].

**Figure 2:**
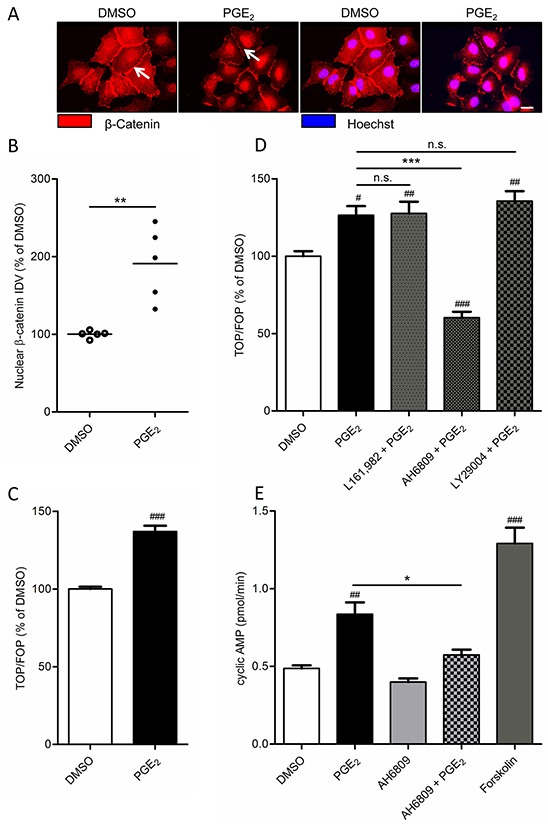
Effect of PGE_2_ on β-catenin nuclear translocation and transcriptional activity in A549 cells **A.** Immunofluorescence images of β-catenin after 18 hours stimulation with PGE_2_. White arrows indicate areas of cell-cell contact. Presence of β-catenin at these areas is decreased in PGE_2_-treated cells, whereas nuclear localization is increased. Scale bar represents 40 μm. **B.** Quantification of β-catenin nuclear localization. Each points represents the average integrated density value (IDV) of 20 cells. **C.** TCF luciferase gene reporter assay of β-catenin transcriptional activity (TOPFlash) after 18 hours stimulation with PGE_2_. **D.** TOPFlash assay of cells co-incubated with antagonists of the EP_4_ receptor (L161,982; 3 μM), the EP_1_ and EP_2_ receptor (AH6809; 10 μM) or PI3 kinase (LY29004, 50 μM). **E.** Measurement of cyclic AMP production. Cells were co-treated with IBMX to inhibit phosphodiesterase activity. Forskolin (10 μM) was used as a positive control for cyclic AMP production by adenylyl cyclase. Data represent mean ± SEM of 5-9 separate experiments. # p < 0.05, ## p < 0.01, ### p < 0.001 compared to DMSO treated cells. * p < 0.05, ** p < 0.01 *** p < 0.001 between the indicated groups.

We have shown in other cell models, that PGE_2_ stabilizes β-catenin [14]. We therefore studied the role of β-catenin in PGE_2_ induced EMT in A549 cells. When we incubated a subconfluent culture of A549 cells with PGE_2_, we observed decreased expression of β-catenin at areas of cell-cell contact (Figure 2A). Accordingly, we observed increased localization of β-catenin within the cell nucleus (Figure 2A-2B), indicating membrane to nucleus translocation of β-catenin. Nuclear β-catenin is a well-known co-activator of several transcriptional programs that are involved in cancer. Nuclear β-catenin activates gene expression primarily through associating with the TCF/Lef family of transcription factors. To assess β-catenin-dependent transcription in response to PGE_2_, A549 cells were transfected with a TCF luciferase reporter gene (TOPFlash) containing multiple TCF binding sites or with a reporter deficient in TCF binding sites (FOPFlash). PGE_2_ induced TCF reporter gene expression, as indicated by an increased TOP/FOP ratio (Figure 2C).

PGE_2_ exerts its effects through activation of E-type prostanoid receptors. A549 cells mainly express the EP_2_ and EP_4_ receptor subtype which are Gα_s_-coupled and enhance formation of intracellular cyclic AMP. To determine which receptor is involved in PGE_2_-induced β-catenin-dependent transcription, EP receptors were blocked using the EP_4_ antagonist L-161,982 and the EP_2_ antagonist AH6809. AH6809 completely prevented PGE_2_-induced β-catenin-dependent transcription while L-161,982 had no effect (Figure 2D). In addition, PI3 kinase, an important lipid kinase which can be activated downstream of EP receptors, also contributes to β-catenin stabilization [12]. However, pharmacological inhibition of PI3 kinase using LY29004 had no effect on PGE_2_-induced β-catenin-dependent transcription in A549 cells, demonstrating that the effects of PGE_2_ are mediated through the Gα_s_-coupled EP_2_ receptor and cyclic AMP. Indeed, when we stimulated cells with PGE_2_, we observed enhanced cyclic AMP synthesis, which was attenuated by pretreatment with AH6809 (Figure 2E).

### Epac1 is required for PGE_2_-induced β-catenin activation

**Figure 3:**
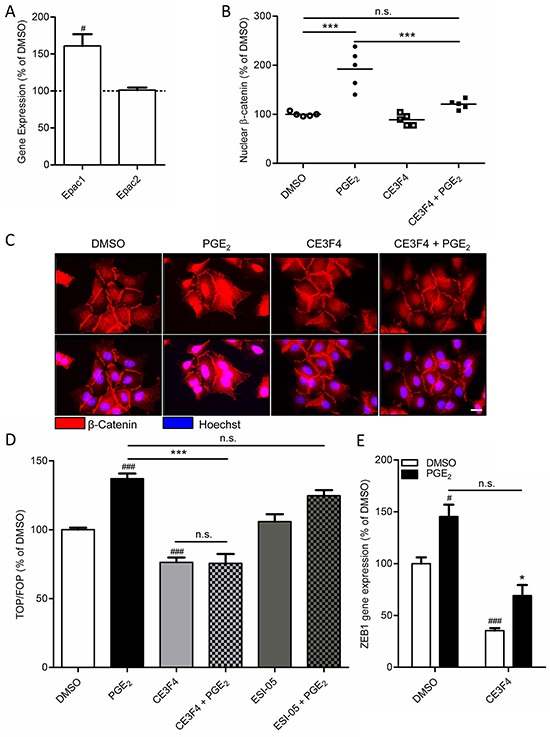
Role of Epac1 in PGE_2_-induced β-catenin nuclear translocation in A549 cells **A.** Gene expression of Epac1, but not Epac2, is increased by PGE_2._ **B.** Quantification of β-catenin nuclear localization. Each points represents the average integrated density value (IDV) of 20 cells. **C.** Immunofluorescence images of β-catenin in cells treated with PGE_2_. Co-incubation with a specific Epac1 antagonist (CE3F4, 20 μM) abolished PGE_2_-induced β-catenin nuclear translocation. Scale bar represents 40 μm. **D.** TOPFlash assay of cells co-incubated with specific antagonists for Epac1 (CE3F4) and Epac2 (ESI-05; 10 μM). **E.** PGE_2_-induced gene expression of ZEB1 is attenuated by co-incubation with CE3F4. Data represent mean ± SEM of 5-9 separate experiments. # p < 0.05, ### p < 0.001 compared to DMSO treated cells. *** p < 0.001 between the indicated groups.

**Figure 4:**
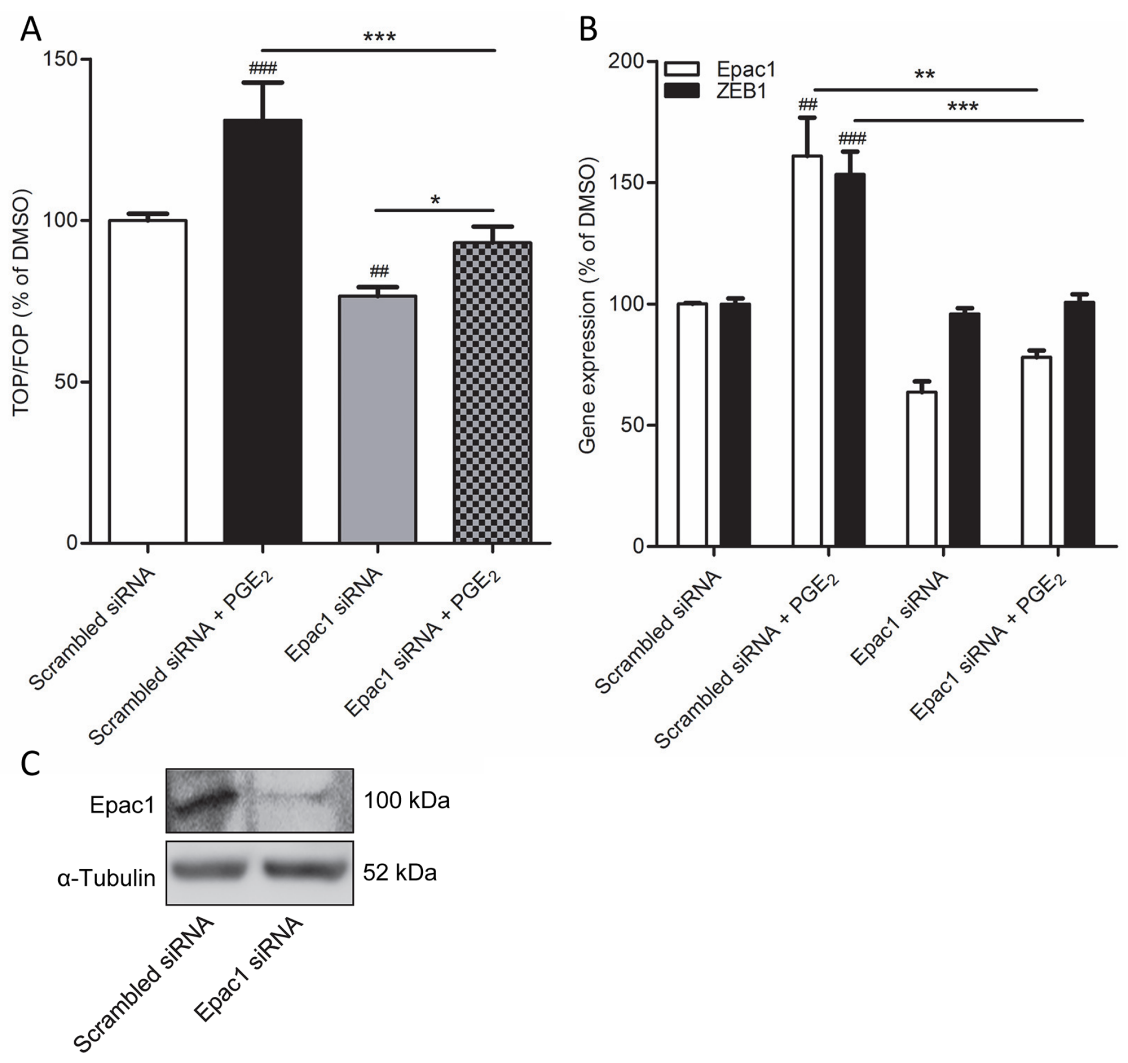
Epac1 knockdown prevents PGE_2_-induced β-catenin transcriptional activity in A549 cells **A.** TOPFlash assay of cells transfected with non-targeting siRNA or Epac1 siRNA for 48h and subsequent PGE_2_ treatment. **B.** Gene expression of Epac1 and ZEB1 in cells transfected with Epac1 siRNA in combination with PGE_2_ treatment. **C.** Knockdown of Epac1 protein in Epac1 siRNA transfected cells. Data represent mean ± SEM of 5-9 separate experiments. ## p < 0.01, ### p < 0.001 compared to non-targeting siRNA transfected cells. ** p < 0.01, *** p < 0.001 between the indicated groups.

Importantly, while expression of the cyclic AMP effector Epac1 was relatively low under basal conditions, we observed that cells undergoing PGE_2_-induced EMT displayed increased expression of Epac1 (Figure 3A). Expression of Epac2 was unaltered. We therefore questioned if Epac1 is involved in PGE_2_-induced β-catenin nuclear translocation and transcriptional activity. To this aim, we used the Epac1 subtype specific inhibitor CE3F4 [37]. PGE_2_-induced β-catenin nuclear translocation was significantly decreased in cells co-incubated with CE3F4, indicating that Epac1 is required (Figure 3B-3C). In accordance with this observation, we demonstrated that PGE_2_-induced β-catenin-dependent transcription was completely abolished in cells incubated with CE3F4, while the Epac2 subtype specific inhibitor ESI-05 [38], had no effect (Figure 3D). Furthermore, PGE_2_-induced expression of the β-catenin target gene and EMT regulator, ZEB1, was attenuated in cells incubated with CE3F4 (Figure 3E). To confirm these observations we applied a siRNA approach to knock down Epac1 expression in A549 cells. PGE_2_-induced β-catenin-dependent transcription was significantly impaired in Epac1 siRNA transfected cells, compared to non-targeting siRNA transfected cells (Figure 4A). In Epac1 siRNA transfected cells, PGE_2_ was no longer able to increase Epac1 expression (Figure 4B-4C). Accordingly, PGE_2_-induced expression of ZEB1 was also abolished in Epac1 siRNA transfected cells (Figure 4B). Collectively, these findings indicate that Epac1 is required for PGE_2_-induced β-catenin-dependent transcription.

### Epac1 is required for PGE_2_-induced cell migration

**Figure 5:**
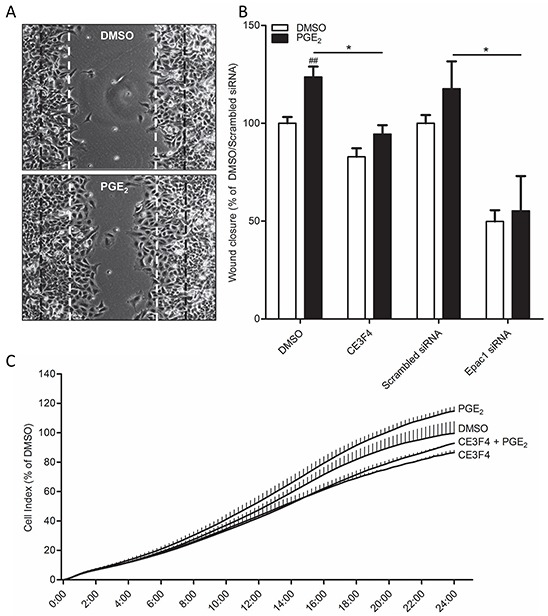
Role of Epac1 in PGE_2_-induced cell migration of A549 cells **A.** Representative images of a wound healing assay 24 hours post scratch. The black lines indicate borders of scratch on 0 hours. The white lines indicate borders of scratch on 24 hours of DMSO treated cells. **B.** Quantification of wound closure of PGE_2_ treated cells in co-incubation with the Epac1 antagonist CE3F4 or in Epac1 silenced cells. **C.** xCELLigence assay of real-time cell migration of cells stimulated with PGE_2_ (left panel) or in co-incubation with the Epac1 antagonist CE3F4. *x*-Axis indicates time in hours. Data represent mean ± SEM of 6 separate experiments. ## p < 0.01 compared to DMSO treated cells. * p < 0.05 between the indicated groups.

Functionally, cells undergoing EMT show increased migratory behavior. When we created a wound area in a confluent monolayer by carefully scratching the monolayer, cells stimulated with PGE_2_ started migrating inwards the wounded area (Figure 5A). Cells that were on the leading edge showed a different, more mesenchymal phenotype, when compared to cells embedded in the monolayer, which appeared epithelial. This confirms our earlier observations in which we found that PGE_2_ only affects cells that are on the leading edge (Figure 1C-1D). In cells co-treated with the Epac1 inhibitor CE3F4 or cells transfected with Epac1 siRNA, PGE_2_ was no longer able to promote migration (Figure 5B). Additionally, in a real time assay for cell migration using the xCELLigence platform [42], PGE_2_ increased migration of cells, which was abolished in cells co-treated with CE3F4 (Figure 5C).

### Nuclear pore localization of Epac1 is required for PGE_2_-induced β-catenin activation

**Figure 6:**
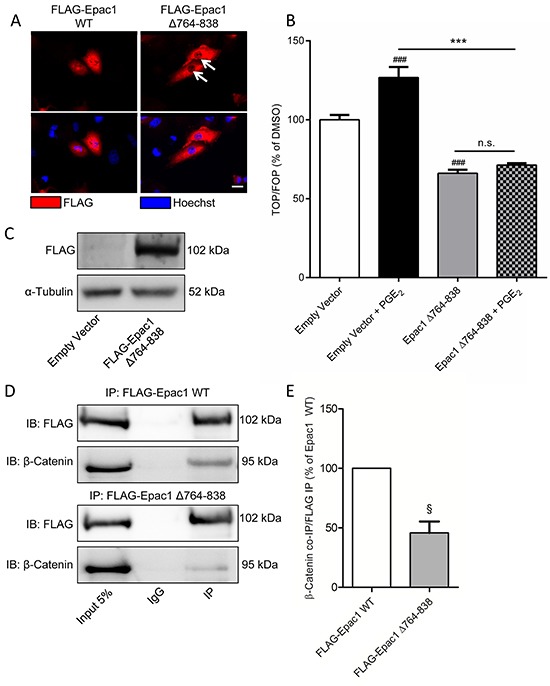
Expression of a mutant Epac1 with a deletion of the 764-838 domain has aberrant localization and prevents PGE2-induced β-catenin transcriptional activity in A549 cells **A.** Immunofluorescence images of FLAG-tagged Epac1 wildtype (WT) and Epac1 Δ764-838. White arrows indicate absence of nuclear localization for the Epac1 deletion mutant. Scale bar represents 40 μm. **B.** TOPFlash assay of cells transfected with Epac1 Δ764-838 and subsequent PGE_2_ treatment. **C.** Western blot image confirming successful expression of FLAG-tagged Epac1 Δ764-838. **D.** Western blot images of a FLAG immunoprecipitation showing co-immunoprecipitation of Epac1 and β-catenin in FLAG-Epac1 WT transfected cells, which is attenuated in and FLAG-Epac Δ764-838 transfected cells. For input we used 5% of the amount of protein used for co-immunoprecipitation. **E.** Quantification of β-catenin co-immunoprecipitation with FLAG immunoprecipitation. β-Catenin was normalized for the amount of FLAG immunoprecipitated. Data represent mean ± SEM of 3-9 separate experiments. # p < 0.05, ### p < 0.001 compared to empty vector transfected cells. ^§^ p < 0.05 compared to FLAG-Epac1 WT transfected cells. *** p < 0.001 between the indicated groups.

Epac1 has been shown to translocate to perinuclear regions following activation by cyclic AMP [26–29]. The nuclear localization of Epac1 requires the catalytic domain of Epac1 binding to the nuclear pore protein RanBP2. When we overexpressed wildtype Epac1 (Figure 6C), we also found a clear nuclear localization of Epac1 (Figure 6A). In contrast, expression of Epac1 with a deletion mutation in the catalytic domain (Δ764-838) renders the mutant Epac1 unable to correctly localize to the nucleus (Figure 6A). As β-catenin also needs to translocate to the nucleus in order to activate β-catenin-dependent transcription, we hypothesized that correct localization of Epac1 is required for nuclear translocation of β-catenin. Indeed, cells expressing Epac1 Δ764-838 did not show PGE_2_-induced β-catenin-dependent transcription (Figure 6B). Interestingly, when we performed a co-immunoprecipitation to confirm a molecular interaction between Epac1 and β-catenin, we found that Epac1 co-immunoprecipitated with β-catenin, but that this interaction was almost absent in cells transfected with the mutant Epac1 Δ764-838 (Figure 6D-6E). This indicates that the interaction between Epac1 and β-catenin may only occur when both proteins are correctly localized to the nucleus. Accordingly, PGE_2_ no longer increased the expression of ZEB1 in cells transfected with the mutant Epac1 Δ764-83 (Figure 6E).

### Ezrin links Epac1 to β-catenin during PGE_2_-induced β-catenin activation

**Figure 7:**
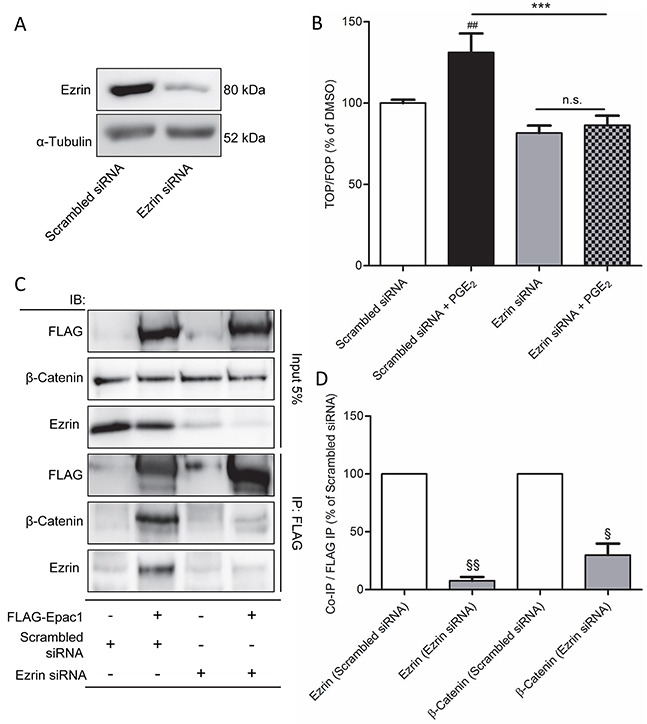
Ezrin knockdown prevents PGE_2_-induced β-catenin transcriptional activity in A549 cells **A.** Knockdown of Ezrin protein in Ezrin siRNA transfected cells. **B.** TOPFlash assay of cells transfected with non-targeting siRNA or Ezrin siRNA for 48 hours and subsequent PGE_2_ treatment. **C.** FLAG co-immunoprecipitation of Epac1, β-catenin and Ezrin in FLAG-Epac1 WT transfected and Ezrin silenced cells. For input we used 5% of the amount of protein used for co-immunoprecipitation. **D.** Quantification of β-catenin and Ezrin co-immunoprecipitation with FLAG immunoprecipitation. β-Catenin and Ezrin were normalized for the amount of FLAG immunoprecipitated. Data represent mean ± SEM of 3-9 separate experiments. ## p < 0.01 compared to non-targeting siRNA transfected cells. ^§^ p< 0.05, ^§§^ p < 0.01 compared to scrambled siRNA transfected cells. *** p < 0.001 between the indicated groups.

Direct binding of Epac1 and β-catenin has not been described before. Nonetheless, the A-kinase anchoring protein family member Ezrin is a scaffold protein which has been shown to bind E-cadherin, β-catenin and Epac1 [23, 43]. In addition, Ezrin is involved in migration of carcinoma cells, including NSCLC cells [24, 44–46]. Therefore, we questioned whether Ezrin might be a common adaptor protein that mediates the interaction between β-catenin and Epac1 in cells undergoing PGE_2_-induced EMT. To study the role of Ezrin, we applied a siRNA knockdown approach (Figure 7A). In Ezrin siRNA-treated cells, PGE_2_-induced β-catenin-dependent transcription was abolished (Figure 7B). In addition, we observed co-immunoprecipitation of Ezrin and Epac1 (Figure 7C-7D). Cells treated with Ezrin siRNA, co-immunoprecipitation of Epac1 and β-catenin was no longer observed, indicating that Ezrin is required for the association of Epac1 and β-catenin.

## DISCUSSION

The contribution of PGE_2_ to EMT in NSCLC cells has previously been reported [4–8]. Our findings confirm that PGE_2_ induced a marked downregulation of E-cadherin and activation of β-catenin, primarily at cells on the migrating edge, subsequently leading to cell migration. It has been reported that PGE_2_ increases levels of stabilized β-catenin [5, 7, 8] and activation of β-catenin-dependent transcription [5]. In addition, emphasizing the importance of β-catenin in PGE_2_-induced EMT in NSCLC cells, pharmacological inhibition of β-catenin with the FH535 compound has been shown to completely abolish the effects of PGE_2_ on EMT [7, 8]. EMT is under tight control of a series of transcription factors, including ZEB1, which regulate the expression of proteins involved in cell-cell adhesion, extracellular matrix composition including matrix metalloproteases, and cell migration. In NSCLC cells, PGE_2_ has been shown to increase expression of the EMT regulator ZEB1 [4, 5]. In a recent study, inhibition of β-catenin-dependent transcription renders NSCLC cells unable to migrate [47]. Our observations confirm that expression of ZEB1 is increased by PGE_2_ and coincides with nuclear translocation of β-catenin and activation of β-catenin-dependent transcription.

We and other have previously shown that PGE_2_, through activation of cyclic AMP, is responsible for activating β-catenin, thus making the Gαs-coupled EP_2_ and EP_4_ receptor the most likely candidates [14]. Here, we observed no effect of specific antagonism of the EP_4_ receptor on PGE_2_-induced β-catenin-dependent transcription, whereas antagonism of the EP_2_ receptor completely abolished the effect of PGE_2_. This finding is contrary to earlier work from others who identified the EP_4_ receptor as the main contributor in PGE_2_-induced cell migration of NSCLC cells, possibly through a mechanism involving PI3 kinase and Akt [5, 6]. However, we observed no effect of PI3 kinase inhibition on PGE_2_-induced activation of β-catenin-dependent transcription, indicating that this EP_4_/PI3 kinase pathway was not linked to β-catenin activation in our cell model. A recent study investigating the effect of PGE_2_ on normal bronchial epithelial cells, found that PGE_2_ induces migration in pre-EMT cells, while in post-EMT cells, PGE_2_ decreases migration [41]. This study showed that this change in response to PGE_2_ was accompanied by a reduction in expression of EP_2_ and EP_4_ receptors in post-EMT. Specific agonism of the EP_2_ and EP_4_ receptor mimicked the effects of PGE_2_, confirming our findings that the Gαs-coupled EP receptors are crucial for PGE_2_-induced EMT.

Earlier studies by us and others have indicated that PGE_2_ activates β-catenin-dependent transcription via cyclic AMP [13, 14, 48–52]. When we investigated expression of the cyclic AMP effector Epac, we observed increased expression of the Epac1 subtype by PGE_2_. Similar to our findings, studies on prostate cancer cell lines [53], squamous cell carcinoma [54] and umbilical cord derived mesenchymal stem cells [55] have also reported increased expression of Epac1 in response to PGE_2_. The role of Epac in regulation of migration and the adherens junction in lung has recently been extensively described in extensive reviews [22, 56]. Importantly, several recent studies have implicated Epac1 in the activation of cancer cell migration and metastasis [35, 36, 57]. In addition, using specific pharmacological inhibitors, it was demonstrated that Epac1 is required for migration and metastasis of pancreatic cancer, both *in vitro* [32, 58] and *in vivo* [30].

We observed that specific inhibition of Epac1 using the pharmacological inhibitor CE3F4 [37] not only decreased migration of NSCLC cells, but also abolished PGE_2_-induced nuclear accumulation of β-catenin, activation of β-catenin-dependent transcription and expression of the EMT regulator ZEB1. Using Epac1 siRNA, we were able to confirm that these observations were due to Epac1 inhibition and not due to off-target effects of the compound.

Epac1 can directly bind to the nuclear pore protein RanBP2 at the nuclear membrane [26–29]. The recent identification of the sequence in the CDC25-HD domain of Epac1 responsible for the binding [28] provided us with a tool to study the role of Epac1 localization to the nuclear pore in PGE_2_-induced activation of β-catenin. In this study we were able to show that blocking Epac1 localization to the nuclear pore, using an Epac1 mutant that lacks this sequence [28], abolished PGE_2_-induced activation of β-catenin-dependent transcription. Studies on the activity of Epac1 binding to RanBP2 have provided contradictory results. One study showed decreased activity [28], while another showed increased activity [26]. Thus, the exact role of Epac1 at the nuclear envelope or nuclear pore is still controversial and warrants further exploration. Additionally, we observed co-immunoprecipitation between Epac1 and β-catenin, a process attenuated in cells expressing the nuclear pore-deficient Epac1 mutant, indicating that the interaction between Epac1 and β-catenin likely occurs at the nuclear pore complex. However, direct interaction between Epac1 and β-catenin remained questionable. Thus, we hypothesizes that an adaptor protein brings together Epac1 and β-catenin. One likely candidate adaptor protein is Ezrin, which has been shown to bind E-cadherin, β-catenin [59] and Epac1 [23]. Importantly, Ezrin depleted NSCLC cells did not show cell spreading in response to Epac1 activation, indicating that Ezrin is critical in this process [24]. Ezrin can be phosphorylated by a plethora of kinases, including PKA which phosphorylates Ezrin on Thr567, thereby promoting an open conformation [24, 60]. Thus PKA, which is also activated by PGE_2_ downstream of the EP_2_ and EP_4_ receptor can activates Ezrin directly. In addition, the open conformation of Ezrin can bind Epac1 at the N-terminal region [23, 24]. Ezrin expression correlates to an invasive phenotype in several carcinomas, including lung carcinoma, and *in vitro*, knockdown of Ezrin reduced the proliferation, migration, and invasion of cancer cells [44–46, 61–63]. Further, reduced cytosolic β-catenin and increased E-cadherin were observed which was restored by Ezrin knockdown. Together, this led us to believe that Ezrin could be the factor bringing together Epac1 and β-catenin during PGE_2_-induced EMT in NSCLC cells. Indeed, knockdown of Ezrin using siRNA strongly reduced PGE_2_-induced β-catenin-dependent transcription and abolished co-immunoprecipitation of Epac1 and β-catenin, indicating that indeed Ezrin is required for the Epac1 and β-catenin association in NSCLC cells.

The critical role of PGE_2_ and β-catenin in tumorigenesis has long been recognized. Altered expression and activity of components of cyclic AMP signaling are common in various cancers. While PKA has received most attention, the role of Epac1 is emerging [31, 64]. Evidence on Epac1 in migration and metastasis is accumulating and this study provides the first clues on the involvement of Epac1 in transducing the signal from PGE_2_ to β-catenin in cancer cell migration. Together with other studies, our current findings indicate that specific targeting of Epac1 could present a novel target in anti-cancer therapy that warrants further exploration. The recent development of specific agonists and antagonists for the Epac isoforms can greatly enhance research on the role of Epac in cancer for the coming years.

## MATERIALS AND METHODS

### Reagents

16,16-dimethyl-PGE_2_, L161,982, AH6809 and Forskolin were from Tocris Bioscience (Bristol, UK). Ly29004 was from Cell Signaling (Beverly, MA). The pharmacological inhibitor for Epac1, CE3F4 was developed by F. Lezoualc'h [37]. The pharmacological inhibitor for Epac2, ESI-05 was developed by X. Cheng [38]. TRIzol® was from Thermo Fisher Scientific (Waltham, MA). All other chemicals were of analytical grade.

### Cell culture

The human alveolar NSCLC cell line A549 was obtained from ATCC (Manassas, VA, USA). Cells were maintained in RPMI 1640 supplemented with 10% v/v heat-inactivated FCS and antibiotics (penicillin 100 U/ml, streptomycin 100 μg/ml) in a humidified atmosphere of 5% (v/v) CO_2_ at 37°C. Cells were washed with HBSS (400 mg/l KCl, 60 mg/l KH_2_PO_4_, 8 g/l NaCl, 350 mg/l NaHCO_3_, 50 mg/l Na_2_HPO_4_.H_2_O, 1 g/l glucose, pH 7.4), dissociated from plates with trypsin/EDTA and seeded in appropriate cell culture plates. Cells were maintained at subconfluence but were allowed to achieve sufficient confluence to allow the formation of epithelial sheets. Cells were maintained in 0.5% (v/v) FCS 24 hours before, and during, stimulation, since 0% (v/v) FCS has been shown to increase production of PGE_2_ in A549 cells [39].

### Transfection

Cells were grown to 60% confluence and were then transfected using TransIT-X2 transfection reagent (Mirus Bio, Madison, WI) in a 2:1 reagent:DNA ratio in complete growth medium. Cells were transfected with plasmid DNA (Per Ø 10 cm dish: 4.5 μg TOPFlash, 4.5 μg FOPFlash, 0.5 μg renilla luciferase (Upstate Biotechnology, Charlottesville, VA), 5.0 μg pFLAG-CMV2-Epac1 WT, 5.0 μg pFLAG-CMV2-Epac1 Δ764-838 or siRNA (50 nM Epac1 siRNA, 50 nM Ezrin siRNA (ON-TARGET Plus SMARTpool, Dharmacon GE, Lafayette, CO).

### TOPFlash assay

β-Catenin dependent transcriptional activity was assayed using the TCF-dependent luciferase reporter TOPFlash. TOPFlash or its negative control FOPFlash transfected cells were subjected to PGE_2_ stimulation for 24 hours, after which luciferase activity was assayed using the Dual Reporter luciferase assay system (Promega, Madison, WI). Transfection with renilla luciferase was used as a transfection and loading control. During transfection with TOPFlash or FOPFlash plasmids, growth medium contained 0.5% FCS because higher concentrations induce nuclear translocation of β-catenin.

### Immunofluorescence

Cells were grown on LabTek II Chamber Slides (Thermo Fisher Scientific, Marietta, OH, USA) and then fixed with 4% (w/v) PFA/4% (w/v) sucrose and permeabilized with 0.3% (w/v) Triton X-100 in cytoskeletal buffer (10 mM Tris, 150 mM NaCl, 5 mM EGTA, 5 mM MgCl_2_, 5 mM glucose, pH 6.1). Cells were then blocked using 1% (w/v) BSA and 2% (v/v) donkey serum in CytoTBS-T (20 mM Tris, 154 mM NaCl, 2 mM EGTA, 2 mM MgCl_2_, 0.1% v/v Tween-20, pH 7.2). β-Catenin, E-cadherin, Vimentin and N-cadherin antibodies were applied overnight after which, secondary FITC-conjugated donkey anti-rabbit IgG or Cy3-conjugated donkey anti-mouse IgG (Jackson Laboratories, Bar Harbor, ME, USA) were applied for 3h. Cell nuclei were visualized with 1 μg/ml Hoechst 33342 (Invitrogen, Carlsbad, CA, USA). Slides were mounted with ProLong® Gold Antifade Reagent (Life Technologies, Marietta, OH, USA). Images were captured with a Leica DM4000 B Fluorescence microscope (Leica Microsystems, Wetzlar, Germany) equipped with a Leica DFC 345 FX camera.

### Quantification of immunofluorescence

Protein expression was quantified using ImageJ software. 5 immunofluorescence pictures, of areas chosen at random, from each of 4 independent experiments were analyzed. In each picture, signal intensity was measured inside a region of interest (ROI) (ROI: E-cadherin – membrane, N-cadherin – whole cell, vimentin – cytoplasm, β-catenin – nucleus). For β-catenin nuclear accumulation, the ROI was determined by creating a dual channel overlay of β-catenin and Hoechst 33342 staining, selecting only the areas positive for Hoechst 33342 and subsequently splitting the channels in the remaining image to create one image per channel. For each ROI, the integrated density value (IDV) was determined using ImageJ. IDV's were normalized for the average IDV of the control condition.

### Isolation of mRNA and real-time PCR analysis

Total mRNA extraction was performed using TRIzol® extraction and cDNA was then obtained using reverse transcription by AMV Reverse Transcriptase Kit (Promega, Madison, WI). qPCR was performed with the Illumina Eco Personal qPCR System (Westburg, Leusden, The Netherlands). Cycle parameters (30s each) were: denaturation at 94°C, annealing at 60°C and extension at 72°C. Target genes were normalized to the geometric mean of reference genes GAPDH and 18S. Primer sequences are listed in Table 1.

**Table 1:** Primer sequences

Gene	Forward	Reverse
RAPGEF3 (Epac1)	GGAAGAACATGGCAAAGTGG	ATGAGCACTGGAATCTGGTC
RAPGEF4 (Epac2)	AAGAACCATCAGGATGTCCG	TGTGGACTGGAGACAAACTG
ZEB1	CTTAGGACAAAAAGTAGGCG	GAACAGGAATCACAGCATAC
CDH1 (E-cadherin)	TGCCCAGAAAATGAAAAAGG	GTGTATGTGGCAATGCGTTC

### Immunoblotting

Cells were lysed in ice-cold RIPA buffer supplemented with phosphatase and protease inhibitors (1 μg/ml aprotinin, 1 μg/ml leupeptin, 1 μg/ml pepstatin A, 1 mM Na_3_VO_4_, 1 mM NaF, 1.06 μg/ml β-glycerophosphate). Equal amounts of protein were subjected to electrophoresis on polyacrylamide gels and transferred to nitrocellulose membranes. Membranes were blocked with Roti-Block (Carl Roth, Karlsruhe, Germany). Protein expression was determined by specific primary and horseradish peroxidase (HRP)-conjugated secondary antibodies in Tris-buffered saline with 0.1% (v/v) Tween-20. Antibodies used are listed in Table 2. Protein expression was visualized by ECL in the Syngene G:BOX HR iChemi gel documentation system (Syngene; Cambridge, UK). Band intensities were quantified by densitometry using ImageJ software.

**Table 2:** Antibodies

Antibody	Source (catalog number)	Dilution WB	Dilution IF
FLAG	Sigma Aldrich (F3165)	1:2000	1:200
Ezrin	Abcam (ab4069)	1:500	
α-Tubulin	Millipore (05-829)	1:2000	
Epac1	Cell Signaling (4155)	1:500	
E-Cadherin	BD Biosciences (610181)	1:1000	
E-Cadherin	Santa Cruz Bio (sc-7870)		1:50
β-Catenin	BD Biosciences (610153)		1:200
N-Cadherin	BD Biosciences (610921)		1:200
Vimentin	Santa Cruz Bio (sc-32322)		1:50

### Cyclic AMP assay

Cells were pretreated for 30 minutes with 1 mM 3-Isobutyl-1-methylxanthine (IBMX) to inhibit phosphodiesterase-mediated breakdown of cyclic AMP and AH6809. After pretreatment, cells were stimulated with PGE_2_ or Forskolin for 5 minutes. Cyclic AMP synthesis was determined by using the cyclic AMP assay kit from BioTrend Chemicals (Cologne, Germany) according to manufacturer's prescriptions.

### Co-Immunoprecipitation

Cells were lysed in non-denaturing ice-cold lysis buffer (20 mM Tris-HCl, 150 mM NaCl, 1 mM EDTA, 1 mM EGTA, 1% (v/v) Triton X-100, pH 7.6) supplemented with protease inhibitors (1 μg/ml leupeptin, 1 mM PMSF, 1mM Na_3_VO_4_, 1 mM NaF, 1.06 μg/ml β-glycerophosphate). Lysates were centrifuged for 10 min at 14,000x*g* at 4°C and supernatants were pre-cleared with protein A/G agarose beads. Subsequently, 500 μg protein was incubated was 1 μg of primary antibody or control IgG for 16 hours at 4°C with gentle rocking. Subsequently, protein A/G agarose beads were added for an additional 2 hours. Beads were collected by centrifugation and washed 5 times in lysis buffer. Beads were then boiled for 5 min at 95°C in denaturing SDS buffer before SDS-PAGE. Co-immunoprecipitated protein was (semi) quantified by densitometry using ImageJ software. Co-immunoprecipitated proteins were corrected for efficiency of immunoprecipitation by normalizing for immunoprecipitated FLAG.

### Wound assay

A confluent monolayer was gently scratch wounded with a pipette tip in triplicate. After washing away the detached cells, cells were allowed to migrate in the wound area in the absence or presence of PGE_2_ and inhibitors. The wound area was photographed immediately after a scratch and then after 6 hours and 24 hours stimulation. Open wound area relative to non-stimulated cells was quantified using TScratch software [40].

### xCELLigence transwell migration

Label-free, real-time quantification of cell migration was assayed using the xCELLigence transwell migration system using CIM-16 plates (RTCA DP, ACEA Biosciences, San Diego, CA). Briefly, 5% (v/v) FCS growth medium was added as a chemoattractant in the bottom. Serum-free media was added to the top chamber and plates were placed in the system for equilibration. Cells were detached using TrypLE Express enzyme to protect cell surface proteins (Thermo Fischer Scientific, Waltham, MA) and 30,000 cells were added to the top chamber in serum-free media containing PGE_2_ and inhibitors. Cells were allowed to adhere for 30 min at room temperature before being placed in the system for 24 hours in a 5% (v/v) CO_2_ humidified atmosphere at 37°C. The system was set to take a cell index measurement at 5 min intervals.

### Statistics

Data represents means ± SEM, from *n* separate experiments. Normality and equal variance were evaluated by Shapiro-Wilk test and F-test. Statistical significance of differences was evaluated by Student's *t*-test, 1-sample *t*-test or 1-way or 2-way ANOVA followed by Tukey's multiple comparisons test, where appropriate.
